# PVP-Engineered WO_3_/TiO_2_ Heterostructures for High-Performance Electrochromic Applications with Enhanced Optical Modulation and Stability

**DOI:** 10.3390/polym17121683

**Published:** 2025-06-17

**Authors:** Pritam J. Morankar, Rutuja U. Amate, Mrunal K. Bhosale, Chan-Wook Jeon

**Affiliations:** School of Chemical Engineering, Yeungnam University, 280 Daehak-ro, Gyeongsan 712-749, Republic of Korea; pritam.nanoworld@gmail.com (P.J.M.); rutu.nanoworld@gmail.com (R.U.A.); mrunal.snst.1@gmail.com (M.K.B.)

**Keywords:** WO_3_/TiO_2_ heterostructure, optical modulation, amorphous–crystalline interface, coloration efficiency, EC device

## Abstract

In response to escalating global energy demands and environmental challenges, electrochromic (EC) smart windows have emerged as a transformative technology for adaptive solar modulation. Herein, we report the rational design and fabrication of a bilayer WO_3_/TiO_2_ heterostructure via a synergistic two-step strategy involving the electrochemical deposition of amorphous WO_3_ and the controlled hydrothermal crystallization of TiO_2_. Structural and morphological analyses confirm the formation of phase-pure heterostructures with a tunable TiO_2_ crystallinity governed by reaction time. The optimized WTi-5 configuration exhibits a hierarchically organized nanostructure that couples the fast ion intercalation dynamics of amorphous WO_3_ with the interfacial stability and electrochemical modulation capability of crystalline TiO_2_. Electrochromic characterization reveals pronounced redox activity, a high charge reversibility (98.48%), and superior coloration efficiency (128.93 cm^2^/C). Optical analysis confirms an exceptional transmittance modulation (ΔT = 82.16% at 600 nm) and rapid switching kinetics (coloration/bleaching times of 15.4 s and 6.2 s, respectively). A large-area EC device constructed with the WTi-5 electrode delivers durable performance, with only a 3.13% degradation over extended cycling. This study establishes interface-engineered WO_3_/TiO_2_ bilayers as a scalable platform for next-generation smart windows, highlighting the pivotal role of a heterostructure design in uniting a high contrast, speed, and longevity within a single EC architecture.

## 1. Introduction

As the world faces increasing environmental challenges driven by climate change, there is a pressing need to reduce energy consumption across all sectors. Among these, the built environment stands out as a major contributor to global energy demands, with indoor heating, cooling, and lighting together accounting for nearly one-third of total energy use [1]. Conventional glazing systems, which allow the uncontrolled transmission of sunlight and thermal radiation, are particularly inefficient and often contribute to excessive reliance on artificial climate regulation. This inefficiency has prompted the search for smart materials that can adapt to environmental changes in real time [2]. EC technologies, especially smart windows, have emerged as a promising solution to this challenge. By enabling reversible changes in transparency under an applied voltage, EC windows offer dynamic control over the light and heat entering a building. This capability significantly reduces the energy required for indoor climate control while simultaneously enhancing occupants’ comfort. As a result, EC systems are gaining momentum as a practical and scalable approach to creating more energy-efficient and environmentally responsible buildings [3,4].

EC materials function by undergoing reversible optical transitions between transparent and colored states, triggered by electrochemical redox reactions involving the insertion and extraction of ions and electrons. A typical ECD is composed of an EC layer, an ion storage layer, an ion-conducting electrolyte, and transparent conducting electrodes [5]. Upon the application of voltage, ions migrate through the electrolyte, while electrons flow through the external circuit, resulting in a change in optical properties. The performance of such devices, quantified by parameters such as switching time, coloration efficiency, optical contrast, and durability, is strongly influenced by their material properties and the interface quality between layers [1,6].

Among various inorganic EC materials, tungsten trioxide (WO_3_) is widely regarded as a benchmark due to its strong optical modulation, relatively fast switching kinetics, and broad spectral response [7]. However, the long-term operation of pure WO_3_ films is often compromised by ion-trapping effects, a slow reversibility, and structural degradation, all of which contribute to a reduced device efficiency over extended cycling. To address these shortcomings, research has increasingly focused on composite and heterostructure systems that combine the high activity of WO_3_ with secondary materials that can stabilize the interface and improve ion transport dynamics. Titanium dioxide (TiO_2_) has emerged as a highly promising candidate for this purpose. Its excellent chemical stability, high transmittance in the visible range, and favorable ion diffusion characteristics make it an ideal modifier in EC configurations [8]. Although not EC in itself, TiO_2_ can regulate ionic mobility, improve charge transfer characteristics, and suppress degradation phenomena when combined with WO_3_ [9,10,11].

Cai et al. reported TiO_2_@ WO_3_ core/shell nanorod arrays with excellent EC properties, including a 57.2% optical modulation at 750 nm, fast switching (2.4/1.6 s), high coloration efficiency (67.5 cm^2^ C^−1^), and 65.1% retention after 10,000 cycles. The improvements stem from the porous core/shell structure, which enhances ion diffusion and charge transfer [12]. Lv et al. developed TiO_2_/WO_3_/TiO_2_ double-heterojunction films, showing a high stability (a 94.72% modulation after 7000 cycles) and superior coloration efficiency (479.3 cm^2^/C). The enhanced performance is due to UV-assisted ion recovery and efficient charge transfer in the heterojunction structure [13]. Nah et al. demonstrated that TiO_2_- WO_3_ nanotube arrays, grown via the anodization of Ti alloys, significantly enhance EC properties. Even small amounts of WO_3_ (0.2 at%) in the oxide improve the contrast, onset potential, and cycling stability of the nanotube-based devices [14]. Ninh et al. prepared WO_3_-TiO_2_ nanocomposite films using doctor-blade and electrochemical deposition. The films showed improved EC properties, with a coloration efficiency of 55.1 cm^2^ C^−1^ for WO_3_-TiO_2_/ITO, significantly higher than TiO_2_/ITO (34.3 cm^2^ C^−1^). These enhanced properties suggest potential applications for large-area smart windows [15]. Hsu et al. prepared TiO_2_/WO_3_ composite thin films with enhanced EC properties. The porous TiO_2_/WO_3_ film showed an improved reversible coloration and bleaching after heat treatment, demonstrating a better EC performance compared to pure TiO_2_ and WO_3_ films [16]. Zhao et al. developed TiO_2_ nanorod-strengthened WO_3_ nano-trees (TWNTs) as a stable EC pseudocapacitive material. The TWNTs exhibited a high optical modulation (ΔT = 79.5% at 633 nm), excellent cycling stability (77.35% ΔT retention after 10,000 cycles), and high coloration efficiency (443.4 cm^2^·C^−1^), making them promising for multifunctional smart windows and EC energy storage devices [17]. Mishra et al. synthesized TiO_2_–Co_3_O_4_ core/shell nanorod arrays on FTO, showing an enhanced EC and supercapacitive performance with 342 F/g, 140 mF/cm^2^ and a coloration efficiency of ~91 cm^2^/C, along with excellent cyclic stability, making them promising for multifunctional electronic devices [18].

In this study, we present a heterostructure EC system engineered by first depositing a WO_3_ film onto a transparent conductive substrate via electrodeposition, followed by the hydrothermal growth of a TiO_2_ overlayer. The hydrothermal reaction time for TiO_2_ synthesis is precisely controlled to investigate its influence on the structure–property relationship and EC performance of the heterostructure system. Unlike conventional configurations where TiO_2_ serves as a foundational or supporting layer, our architecture places TiO_2_ as a surface interface on WO_3_, forming a functional WO_3_/TiO_2_ (WTi) heterostructure. This design facilitates improved ion diffusion, enhanced electrochemical reversibility, and a better accommodation of charges. The heterostructure structure demonstrates substantial improvements in coloration efficiency, switching speed, and cyclic stability compared to single-layer WO_3_ systems. These findings highlight the potential of rationally designed layered architectures in advancing the performance of ECDs and underscore their relevance in the development of intelligent energy-saving window technologies.

## 2. Experimental Section

### 2.1. Reagents and Materials

All chemicals and solvents used in this study were of analytical grade and used without further purification. Fluorine-doped tin oxide (FTO)-coated glass substrates (sheet resistance ~10 Ω/sq) were procured from the MTI Corporation, South Korea, and used as conductive substrates. Prior to use, the FTO substrates were thoroughly cleaned by sequential ultrasonication in ethanol, acetone, and deionized (DI) water, each for 15 min, followed by drying in air. The main reagents employed included sodium tungstate dihydrate (Na_2_WO_4_·2H_2_O), polyvinylpyrrolidone (PVP, 40,000 g/mol (PVP 40)), titanium (IV) butoxide (C_16_H_36_O_4_Ti, 97%), hydrochloric acid (HCl, 35–37%), hydrogen peroxide (H_2_O_2_, 30%), nitric acid (HNO_3_), lithium perchlorate (LiClO_4_), and propylene carbonate (PC), all purchased from Sigma–Aldrich (USA) and used as received.

### 2.2. Electrodeposition of WO_3_ on FTO Substrates

WO_3_ thin films were deposited onto cleaned FTO glass substrates via a cyclic voltametric electrodeposition method. The electrodeposition process was carried out using a Biologic WBCS3000 electrochemical workstation configured in a three-electrode setup. The FTO substrate served as the working electrode, a platinum wire as the counter electrode, and an Ag/AgCl electrode as the reference. The precursor solution was prepared by dissolving 15 mM Na_2_WO_4_·2H_2_O in 100 mL of DI water. To this solution, 0.3 g of PVP was added and stirred until completely dissolved, acting as a stabilizing and morphology-directing agent. Next, 1 mL of 30% H_2_O_2_ was added under stirring to form a stable yellow-colored peroxotungstate complex. A small amount of HNO_3_ was added dropwise while maintaining the solution temperature at 45 °C to adjust the pH, which reached approximately 1.5, and to further stabilize the precursor species. The solution was then cooled to room temperature before use. WO_3_ films were deposited using cyclic voltammetry in a potential window of ±1 V at a scan rate of 50 mV/s for 20 cycles. Following deposition, the WO_3_-coated FTO substrates were rinsed thoroughly with DI water and ethanol to remove residual species and dried at 60 °C under ambient conditions. These WO_3_ films served as base layers for the subsequent hydrothermal growth of TiO_2_.

### 2.3. Hydrothermal Growth of TiO_2_ on WO_3_ Films

**Figure 1:**
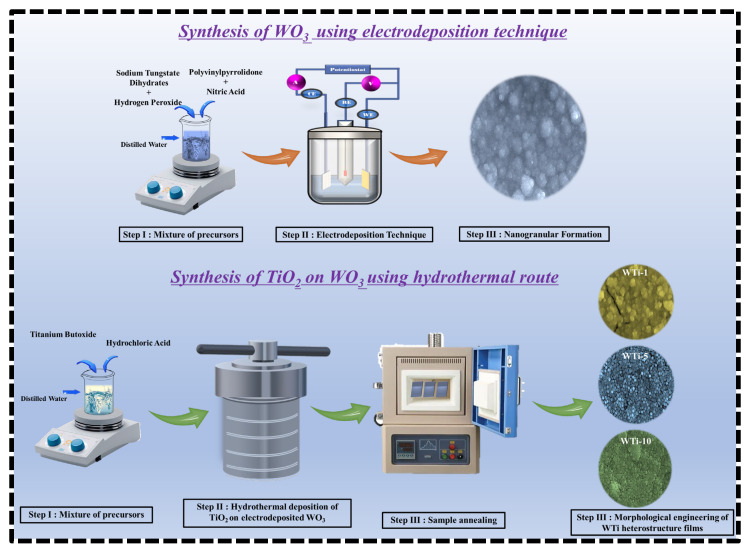
Schematic diagram of the potential process for forming WTi heterostructure thin films.

TiO_2_ films were grown on the pre-deposited WO_3_ layers via a conventional hydrothermal method. The precursor solution was prepared by mixing 50 mL of DI water with 50 mL of concentrated HCl under continuous stirring. After complete mixing, 1 mL of C_16_H_36_O_4_Ti was added dropwise, and the resulting solution was stirred for an additional 30 min at room temperature to ensure homogeneity. The pH of the resulting acidic precursor solution was approximately 1.0. The WO_3_-coated FTO substrates were placed vertically in a Teflon-lined stainless-steel autoclave containing the precursor solution. The autoclave was then sealed and maintained at 150 °C for different reaction times of 1, 5, and 10 h to examine the effect of the duration of TiO_2_ growth on the properties of the heterostructure films. After the hydrothermal process, the autoclave was allowed to cool naturally to room temperature. The resulting TiO_2_-coated WO_3_ films were carefully removed, rinsed thoroughly with DI water to eliminate any unreacted precursor residues, and dried under ambient conditions. To improve the film’s quality and enhance their crystallinity, the samples were annealed in air at 500 °C for 30 min. The obtained WTi heterostructure films were labeled as WTi-1, WTi-5, and WTi-10, corresponding to TiO_2_ growth times of 1, 5, and 10 h, respectively. These samples were used for all subsequent characterizations. Figure 1 illustrates the schematic representation of the fabrication process for the WTi heterostructure.

### 2.4. Electrochromic Device Fabrication

An EC was fabricated using the WTi heterostructure thin film with a TiO_2_ growth time of 5 h (designated as WTi-5) as the working electrode. The device architecture followed a sandwich configuration consisting of glass/FTO/WTi-5 as the active electrode, a liquid electrolyte, and a counter electrode of FTO-coated glass. A 1 M solution of LiClO_4_ in PC served as the electrolyte. The electrolyte was injected between the two electrodes, and the assembly was sealed using a transparent double-sided adhesive film (Scotch Brand Tape, 3M Seoul, South Korea) to prevent leakage and ensure good contact. The effective working area of the ECD was 3 × 4 cm^2^. This device configuration was used for all subsequent optical modulation and electrochemical performance measurements.

## 3. Sample Characterization and Electrochemical Measurements

The crystalline structure and phase purity of the WTi heterostructure thin films were analyzed by X-ray diffraction (XRD) using a PANalytical diffractometer (Almelo, The Netherlands) equipped with Cu-Kα radiation (λ = 1.5406 Å). The surface morphology and elemental distribution were examined through field-emission scanning electron microscopy (FE-SEM, S4800, Hitachi, Tokyo, Japan) combined with energy-dispersive X-ray spectroscopy (EDS). Prior to FESEM and EDS analysis, all samples were coated with a thin platinum layer to improve their surface conductivity and imaging resolution. X-ray photoelectron spectroscopy (XPS, K-Alpha, Thermo Scientific, UK) was employed to investigate the surface chemical composition and oxidation states of the constituent elements. High-resolution transmission electron microscopy (HRTEM, Tecnai F21, FEI Company), coupled with selected-area electron diffraction (SAED), was employed to further probe the intricate structures and interfacial characteristics within the heterostructure. Electrochemical performance was evaluated using a Biologic WBCS3000 battery cycler in a conventional three-electrode configuration, where the WTi-coated FTO served as the working electrode, platinum wire as the counter electrode, and an Ag/AgCl electrode as the reference. A 1 M LiClO_4_ solution in PC was used as the electrolyte for all measurements. The optical transmittance of the electrodes in both the colored and bleached states was recorded using a UV–Vis spectrophotometer (S-3100, SCINCO) coupled to an electrochemical workstation (IVIUM Technologies, COMPACTSTAT, Fujian Province, China) for in situ optical–electrochemical analysis.

## 4. Results and Discussion

### 4.1. X-Ray Diffraction (XRD)

**Figure 2:**
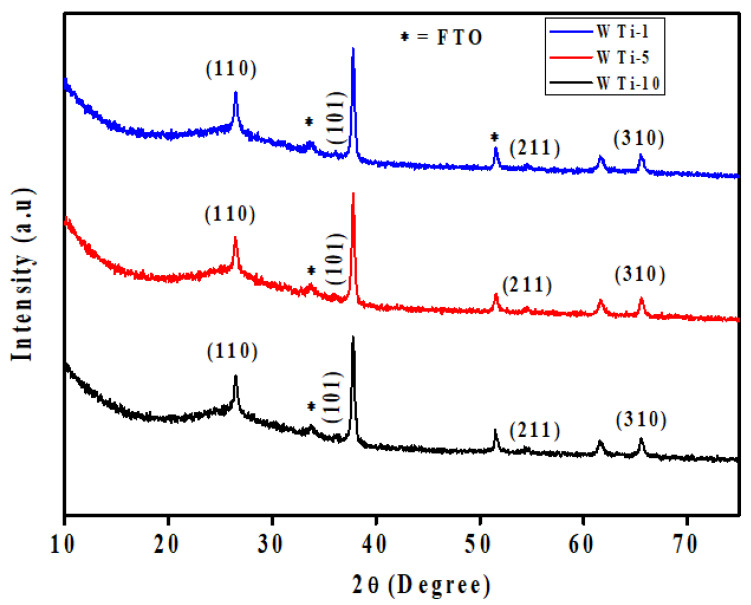
XRD pattern of WTi-1,WTi-5, and WTi-10 heterostructure thin films.

To investigate the structural characteristics of the synthesized films, XRD was performed on the WTi heterostructures fabricated by first depositing a WO_3_ layer onto FTO substrates via electrodeposition, followed by the hydrothermal growth of TiO_2_ for varying durations. The resulting samples, labeled as WTi-1, WTi-5, and WTi-10, correspond to TiO_2_ growth times of 1, 5, and 10 h, respectively. All diffraction profiles, as shown in Figure 2, display a broad and intense hump in the 2θ range of 20–27°, which is characteristic of the amorphous structure of the underlying WO_3_ layer. This broad feature indicates the absence of a long-range crystalline order and is typical for electrochemically deposited WO_3_ [19]. The amorphous nature of this layer is advantageous for EC applications, as it provides enhanced ion diffusion channels, structural flexibility, and abundant active sites for redox reactions. Overlaid on the amorphous background are distinct and sharp peaks at 27.4°, 36.1°, 54.3°, and 69.0°, which correspond to the (110), (101), (211), and (310) planes of rutile TiO_2_. These reflections match well with the tetragonal rutile phase as indexed by JCPDS card no. 01-088-1175, confirming the successful formation of crystalline TiO_2_ on top of the WO_3_ layer. The increasing intensity of these TiO_2_ peaks from WTi-1 to WTi-10 reflects a clear enhancement in crystallinity with longer hydrothermal growth durations. Additional minor peaks, marked with an asterisk (*), originate from the underlying FTO substrate. No extraneous or unidentified peaks are detected, confirming the phase purity of the heterostructures. This combination of a structurally disordered WO_3_ base and a well-crystallized TiO_2_ overlayer forms a highly functional interface, where the amorphous layer supports rapid ion transport while the crystalline layer enhances stability. Such a heterostructured configuration is particularly suited for advanced ECDs, enabling efficient optical modulation and long-term cycling performance [20].

### 4.2. X-Ray Photoelectron Spectroscopy (XPS) Analysis

**Figure 3:**
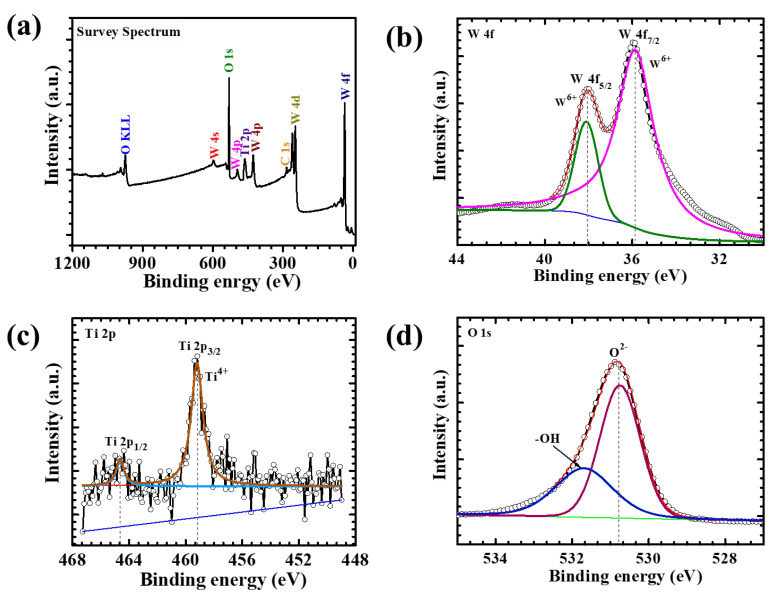
(**a**) XPS survey spectrum, high-resolution (**b**) W 4f spectrum, (**c**) Ti 2p spectrum, and (**d**) O 1 s spectrum of the WTi-5 sample.

To probe the surface chemical composition and electronic states of the WTi heterostructure, XPS was performed on the WTi-5 electrode. The survey spectrum (Figure 3) clearly confirms the presence of tungsten (W), titanium (Ti), and oxygen (O) elements, verifying the successful fabrication of the bilayer heterostructure, with TiO_2_ formed as the top layer over an electrodeposited WO_3_ base. The high-resolution W 4f spectrum (Figure 3b) shows two distinct peaks located at approximately 35.7 eV and 37.8 eV, corresponding to the W 4f_7/2_ and W 4f_5/2_ spin–orbit components, respectively. The energy separation of ~2.1 eV and the symmetric line shape are characteristic of W^6+^ in fully oxidized WO_3_. Notably, no additional shoulders or sub-peaks associated with lower oxidation states (such as W^4+^ or W^5+^) are detected, indicating the chemical stability and purity of the amorphous WO_3_ layer even after subsequent hydrothermal treatment during TiO_2_ deposition [21]. The Ti 2p spectrum (Figure 3c) exhibits two prominent peaks at binding energies of ~458.5 eV (Ti 2p_3/2_) and ~464.2 eV (Ti 2p_1/2_), with a spin–orbit splitting of approximately 5.7 eV. This well-resolved doublet confirms the presence of Ti in the +4-oxidation state, characteristic of stoichiometric TiO_2_. The sharpness and intensity of the peaks, along with the absence of any sub-valent species, suggest the formation of a chemically pure and uniform TiO_2_ overlayer on top of WO_3_.The O 1s core-level spectrum (Figure 3c) features a dominant peak centered at ~530.2 eV, corresponding to lattice oxygen (O^2−^) within the metal oxide matrix. A secondary shoulder at ~531.5 eV is attributed to surface hydroxyl groups and chemisorbed oxygen species, commonly observed in oxide-based thin films. These surface functionalities are known to enhance electrochemical reactivity and facilitate ion transport, thus potentially improving EC performance. Altogether, the XPS analysis confirms the successful formation of a chemically well-defined WO_3_/TiO_2_ heterostructure, with W^6+^ and Ti^4+^ oxidation states and a clear bilayer interface. The compositional purity and oxidation state uniformity across both layers suggest a robust chemical integrity and excellent interfacial compatibility. This synergistic heterostructure design is expected to play a critical role in enhancing redox reversibility, optical modulation, and long-term cycling stability in ECD applications [19,22].

To improve data accessibility, Table 1 summarizes the key XRD and XPS features, including corresponding peak positions, phase assignments, and oxidation states for the WTi heterostructure.

### 4.3. Morphological and Elemental Compositional Characteristics

**Figure 4:**
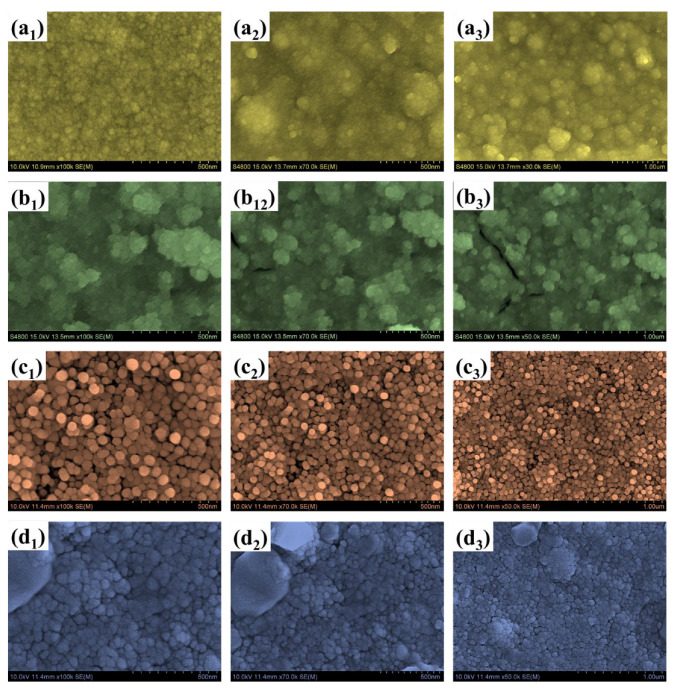
FE-SEM images of (**a_1_**–**a_3_**) bare WO3, (**b_1_**–**b_3_**) WTi-1, (**c_1_**–**c_3_**) WTi-5, and (**d_1_**–**d_3_**) WTi-10 samples (**b**,**d**) EDX spectra of single-layered WO_3_ and W@C bilayer composite thin films.

Field-emission scanning electron microscopy (FESEM) was employed to investigate the morphological evolution of the WTi heterostructure films, synthesized by first electrodepositing a WO_3_ layer onto FTO substrates, followed by the hydrothermal growth of TiO_2_ for varying durations of 1, 5, and 10 h. This sequential deposition process allowed for a detailed evaluation of how the overgrowth of TiO_2_ influences the surface morphology of the underlying WO_3_ and the overall architecture of the bilayer system. The surface morphology of the bare WO_3_ film, shown in Figure 4(a_1_–a_3_), reveals a densely packed nanogranular texture composed of uniformly distributed nanoparticles. While this structure offers a high surface-to-volume ratio, which is generally beneficial for electrochemical activity, it lacks hierarchical complexity and may suffer from a limited structural robustness and suboptimal ion transport over extended cycling. Moreover, the presence of irregular agglomerated domains suggests uneven nucleation, which may hinder effective charge transport and lead to ion-trapping effects during EC switching. When TiO_2_ was deposited hydrothermally for 1 h on the WO_3_ base layer (sample WTi-1), the resulting heterostructure (Figure 4(b_1_–b_3_)) exhibited a less uniform morphology characterized by large, irregular aggregates. These features are attributed to insufficient nucleation and uncontrolled growth during the short hydrothermal process. The poor film continuity and non-uniform coverage increase interfacial resistance and limit efficient ion diffusion, thereby impairing EC performance. In contrast, extending the TiO_2_ growth time to 5 h (WTi-5, Figure 4(c_1_–c_3_)) led to significant improvements in surface morphology. The images reveal a more homogeneous and well-organized nanoparticle structure, with spherical features evenly distributed across the surface. This optimized morphology results from a balanced nucleation-growth regime during the hydrothermal reaction, enabling uniform coverage of TiO_2_ over the WO_3_ base. The resulting surface facilitates enhanced electrolyte access, reduces grain boundary resistance, and promotes faster ion transport, which are all contributing to superior EC performance. However, further increasing the hydrothermal reaction time to 10 h (WTi-10, Figure 4(d_1_–d_3_)) produced an overly compact surface morphology. The densely packed TiO_2_ particles reduced the interparticle voids essential for ion diffusion and electrolytes’ penetration. Although the particle uniformity improved, the excessive grain growth and reduced porosity negatively impacted ionic accessibility and limited the EC switching kinetics. The morphological transitions observed from bare WO_3_ to the WTi-1, WTi-5, and WTi-10 samples are governed by the time-dependent nucleation and growth dynamics of the TiO_2_ hydrothermal process. At shorter durations, rapid nucleation with an insufficient growth time led to poor surface coverage and agglomeration. The 5 h reaction time enabled controlled crystallization and uniform deposition through mechanisms such as Ostwald ripening, resulting in an optimal particle distribution. However, at the longest reaction time (10 h), excessive growth and particle fusion compressed the surface microstructure, reducing active ion transport channels [2,13,23].

**Figure 5:**
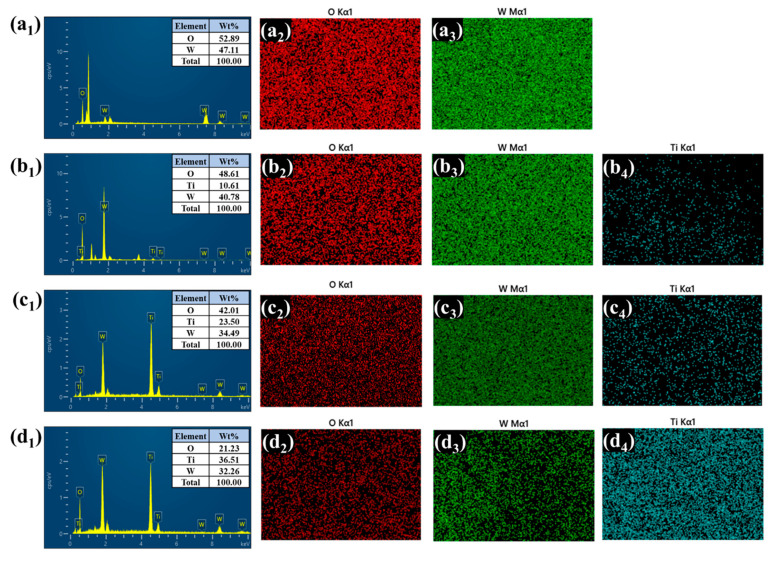
(**a_1_**–**d_4_**) EDS and elemental presentation of bare and all the WTi heterostructure thin films.

Energy-dispersive X-ray spectroscopy (EDS) and elemental mapping further confirmed the presence and spatial distribution of tungsten (W), titanium (Ti), and oxygen (O) in all the heterostructure films depicted in Figure 5(a_1_–d_4_). In the bare WO_3_ sample, only W and O signals were detected, uniformly distributed across the film. After TiO_2_ deposition, the WTi-1, WTi-5, and WTi-10 samples exhibited a clear presence of Ti, along with W and O. Notably, WTi-5 displayed the most homogenous elemental distribution, consistent with the optimized surface morphology observed in the FESEM analysis. In WTi-10, although the Ti content further increased, it was accompanied by dense elemental packing, suggesting reduced electrolyte accessibility. These results indicate that the intermediate TiO_2_ growth time (5 h) offers the best compositional and morphological balance for enhanced EC performance in WTi heterostructure systems.

### 4.4. Transmission Electron Microscopy (TEM) Analysis

**Figure 6:**
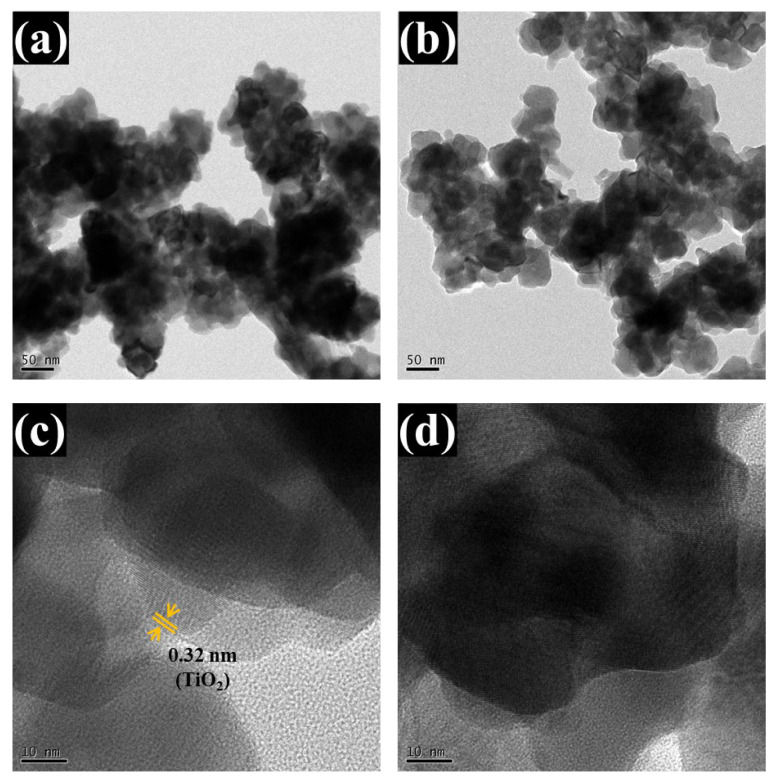
TEM analysis of the optimized WTi-5 thin film: (**a**,**b**) low-magnification images showing spherical nanoparticles, (**c**,**d**) high-resolution images displaying a distinct interface between amorphous WO3 and crystalline TiO_2_ with visible lattice fringes.

The analyzed TEM images (Figure 6a–d) further validate the bilayer heterostructure. Low-magnification TEM images (Figure 6a,b) show spherical nanoparticles forming a continuous network. This observation aligns closely with the FESEM results, which revealed a homogeneous and well-organized nanoparticle structure with distinct spherical features. High-resolution TEM (Figure 6c,d) shows a sharp interface between two layers: the lower amorphous WO_3_ (appearing as a disordered region) and the upper crystalline TiO_2_, which displays distinct lattice fringes. The measured interplanar spacing of ~0.325 nm corresponds to the (110) planes of tetragonal rutile TiO_2_, consistent with the standard JCPDS card no. 01-088-1175. The amorphous nature of WO_3_ is confirmed by the absence of lattice ordering and corroborated by earlier XRD analyses. These results unequivocally confirm the successful formation of a coherent, structurally integrated WO_3_/TiO_2_ heterojunction and strongly support the interface-engineered design strategy.

## 5. Electrochromic Analysis

**Figure 7:**
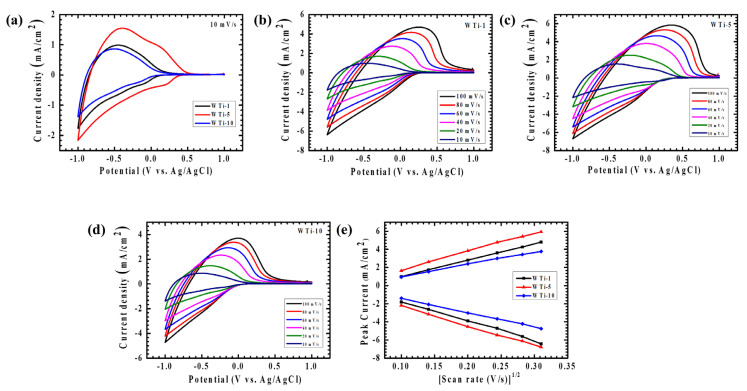
Cyclic voltammetry of (**a**) WTi-1,WTi-5 and WTi-10 heterostructure thin films recorded at a scan rate of 10 mV/s, in a potential window from +1 V to −1 V. Cyclic voltammetry of (**b**) WTi-1, (**c**) WTi-5, (**d**) WTi-10 thin films at different scan rates (10–100 mV/s). (**e**) Plot of peak current vs. (scan rate) 1/2 of all samples for the diffusion coefficient.

The electrochemical characteristics of the WTi heterostructure electrodes were thoroughly explored via cyclic voltammetry (CV) to gain deeper insights into their charge storage dynamics and ion transport behavior at the electrode–electrolyte interface. All measurements were performed using a standard three-electrode setup in 1 M LiClO_4_ dissolved in PC, with the potential window set between +1 V and −1 V versus Ag/AgCl. To evaluate both diffusion-controlled and surface-related electrochemical processes, scan rates were systematically varied from 10 to 100 mV/s. The CV curves for the WTi heterostructure films, as depicted in Figure 7a–d, clearly exhibit distinct and well-defined redox peaks, which are indicative of robust and reversible faradaic reactions taking place during the electrochemical cycles. Specifically, Figure 7a contrasts the CV responses of WTi-1, WTi-5, and WTi-10 at a scan rate of 10 mV/s, while the individual electrochemical signatures of each sample are shown in Figure 7b–d, respectively. The appearance of nearly symmetric anodic and cathodic peaks across all the samples underscores their excellent electrochemical reversibility, ensuring a low polarization and minimal energy dissipation throughout the redox transitions [24,25].

Additionally, the progressive broadening of the CV loops and the consistent increase in current density with rising scan rates point to enhanced ion transport kinetics and an improved charge storage capacity, particularly driven by the facile intercalation and deintercalation of Li⁺ ions Figure 7e. Among all the tested electrodes, the WTi-5 composition demonstrated the highest peak current values, revealing its superior electrochemical response compared to WTi-1 and WTi-10. To further quantitatively elucidate their ion diffusion characteristics, the apparent diffusion coefficients (D) were precisely determined via the Randles–Sevcik Equation (1) [4]:

(1) $$D^{1/2}=\frac{i_{p}}{2.69\times 10^{5}\times n^{3/2}\times A\times C\times v^{1/2}}$$

Here, *n* corresponds to the electron number participating in the redox reactions, *A* is the electroactive surface area, *C* is the concentration of redox-active species, and *v* represents the scan rate. The computed diffusion coefficients at a controlled scan rate of 10 mV/s are detailed as 0.348 × 10^−9^/1.142 × 10^−9^ cm^2^/s, 0.938 × 10^−9^/1.66 × 10^−9^ cm^2^/s, and 0.315 × 10^−9^/0.683 × 10^−9^ cm^2^/s for the WTi-1, WTi-5, and WTi-10 samples corresponding to oxidation/reduction, respectively. Notably, WTi-5 distinctly exhibited the highest diffusion coefficient among the series, underscoring its significantly improved ionic mobility and expedited charge transfer kinetics. This pronounced performance can be directly linked to the optimized structural features of WTi-5—notably, its homogeneous and well-organized nanoparticle structure with spherical features evenly distributed across the surface, which offers abundant electroactive sites and short diffusion pathways for Li⁺ ions. In contrast, WTi-1 exhibited rapid nucleation with an insufficient growth time, leading to poor surface coverage and agglomeration. On the other hand, WTi-10 suffered from excessive growth, and particle fusion compressed the surface microstructure, reducing active ion transport channels. The exceptional charge storage capability and fast redox switching behavior exhibited by WTi-5 can therefore be attributed to its ideal balance of uniformity and structural connectivity. These features collectively ensure improved ion accessibility and efficient charge transport, positioning the WTi-5 electrode as the most promising candidate for high-performance EC applications [26].

**Figure 8:**
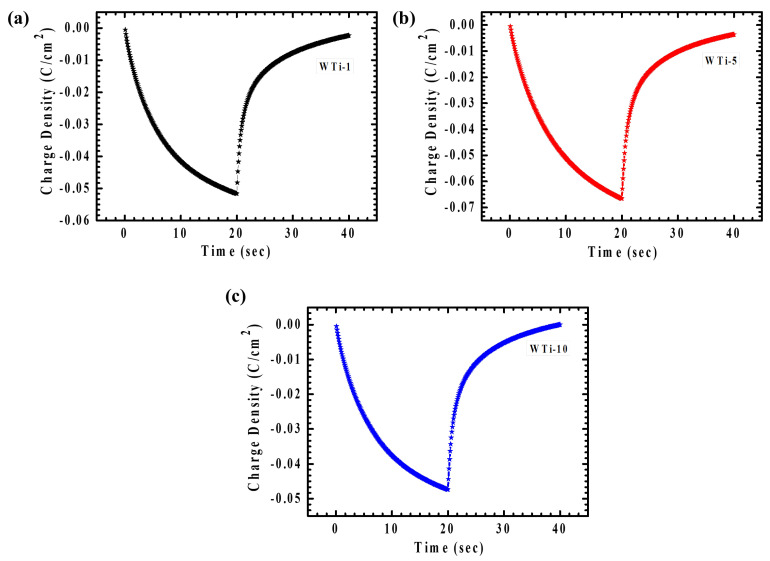
(**a**–**c**) CC plot of WTi-1,WTi-5, and WTi-10 heterostructure thin films.

Chronocoulometry (CC) is an effective approach for monitoring the quantity of ions inserted or extracted during a defined time span under an applied potential. In this work, CC was applied to assess the influence of various TiO_2_ reaction times on charge transfer behavior. Figure 8a–c displays the EC responses of the WTi heterostructure films in both colored and bleached conditions, measured across a potential range from +1 V to −1 V over a period of 40 s. One important parameter obtained from the CC is electrochemical reversibility, representing the proficiency of ion intercalation and deintercalation (2) [4,27].

(2) $$Reversibility=\frac{Q_{di}}{Q_{i}}\times 100$$

Here, *Q_i_* and *Q_di_* represent the amounts of charge intercalated and deintercalated, respectively. Table 2 summarizes the reversibility values for the WTi heterostructure electrodes, confirming good reversibility across all the samples. Among them, the WTi-5 sample showed outstanding reversibility (98.48%), underscoring the crucial influence of its well-optimized surface morphology in providing numerous electroactive sites, thereby promoting efficient ion insertion and extraction. In contrast, the WTi-1 and WTi-10 films exhibited a lower reversibility, primarily due to Li⁺ ion trapping during the electrochemical process, which can be attributed to their aggregated and densely packed surface structures [28].

The enhancement in EC performance observed in the WTi-5 heterostructure is strongly linked to the interfacial synergy between the amorphous WO_3_ base layer and the crystalline TiO_2_ overlayer. TiO_2_, although EC itself, plays a critical role as a conductive and electrochemical buffer [14,17]. The crystalline rutile TiO_2_ phase grown hydrothermally on top of WO_3_ introduces a more stable and continuous electron transport pathway due to its relatively higher electronic conductivity and structural rigidity. This acts to suppress the charge localization and mitigate ion-trapping effects commonly associated with amorphous WO_3_ films. Moreover, the TiO_2_ overlayer helps in modulating the interfacial electric field, which enhances Li^+^ intercalation and deintercalation dynamics by reducing energy barriers at the electrolyte/electrode interface. The improved surface morphology and reduced grain boundary resistance also contribute to a lower charge-transfer resistance, as evidenced by the more pronounced and reversible redox peaks in the CV profiles of WTi-5. Together, these effects confirm that TiO_2_ not only reinforces the structural integrity of the EC film but also actively facilitates ionic mobility and charge transport, leading to an enhanced coloration efficiency, faster switching kinetics, and superior long-term cycling stability [24,25,29].

**Figure 9:**
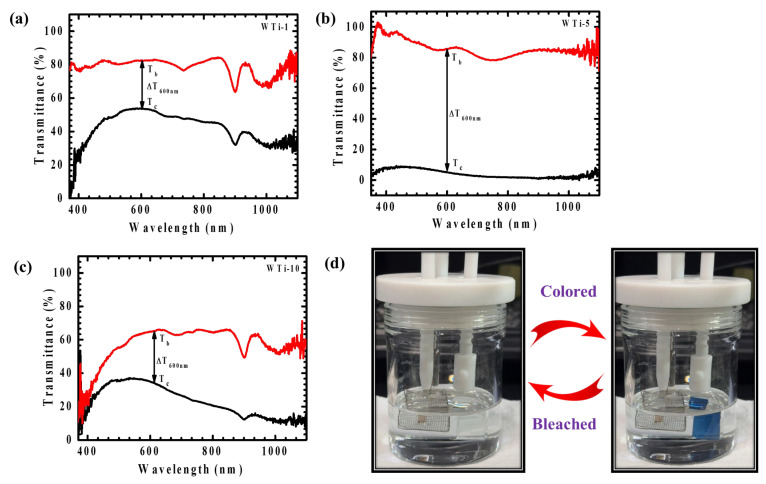
(**a**–**c**) Transmittance spectra of WTi-1,WTi-5, and WTi-10 heterostructure thin films; (**d**) photographs of bleached and colored states of WTi-5 thin film.

The EC performance of WTi heterostructure thin films was systematically assessed through in situ transmittance measurements in both colored and bleached states, spanning the visible spectral range (350–1100 nm), as depicted in Figure 9a–c. Initially, all samples exhibited a high transparency within the visible region. Upon applying a negative potential of −1 V, the films underwent coloration, whereas a positive potential of +1 V restored their transparency through the bleaching process. The coloration phenomenon is attributed to the simultaneous insertion of ions and electrons into the electrode matrix, leading to the formation of polaronic states that absorb visible light and reduce transmittance. Optical modulation (ΔT), defined as the difference between the bleached (T_b_) and colored (T_c_) transmittance values (ΔT = T_b_ − T_c_), serves as a key parameter to evaluate EC efficiency. Table 2 presents the ΔT values, along with the corresponding T_b_ and T_c_ percentages, for the WTi heterostructure films. Among all the samples, WTi-5 exhibited the highest optical modulation, reaching 82.16% at 600 nm, significantly exceeding that of WTi-1 (31.07%) and WTi-10 (30.58%). This highlights WTi-5 as the most effective sample for optical switching behavior. In its colored state, WTi-5 demonstrated a deep blue appearance with a transmittance as low as 4.16%, in contrast to the higher transmittance values recorded for WTi-1 (53.02%) and WTi-10 (35.47%). The superior EC response of the WTi-5 thin film can be ascribed to its well-organized surface morphology, which offers shorter ion diffusion paths and an improved electrode–electrolyte interaction, thereby enhancing ion insertion/extraction dynamics. The photographs of the bleached and colored states of WTi-5, shown in Figure 9d, further substantiate its enhanced EC performance. Conversely, WTi-1 and WTi-10 exhibited a relatively inferior optical modulation, likely due to their less favorable surface morphologies, characterized by particle agglomeration and surface defects, which impede ion transport and limit EC efficiency [3,28].

Coloration efficiency (CE) is a key parameter in assessing EC performance, representing how effectively optical modulation (quantified as the change in optical density, ΔOD) occurs relative to the amount of charge inserted at a specific wavelength. It is mathematically defined as (3)

(3) CE=ΔODQiA

Here, *A* represents the active surface area of the electrode, and *Q_i_* denotes the amount of charge intercalated. The calculated CE values for all thin films at 600 nm are summarized in Table 2. Among them, the WTi-5 film exhibited the highest CE of 128.93 cm^2^/C. This elevated value is particularly advantageous for EC applications, as it signifies the material’s capability to induce a substantial optical contrast with minimal ion and electron involvement. This efficiency correlates with the superior performance of the WTi-5 sample, which provides a dense distribution of electroactive sites, enhancing ion diffusion kinetics. Furthermore, the film’s reduced internal resistance lowers the energy requirement for coloration, thus optimizing the switching response and improving its overall efficiency. These attributes highlight the excellent EC behavior of the WTi-5 thin film, positioning it as a strong candidate for real-world applications such as energy-saving smart windows [19].

**Figure 10:**
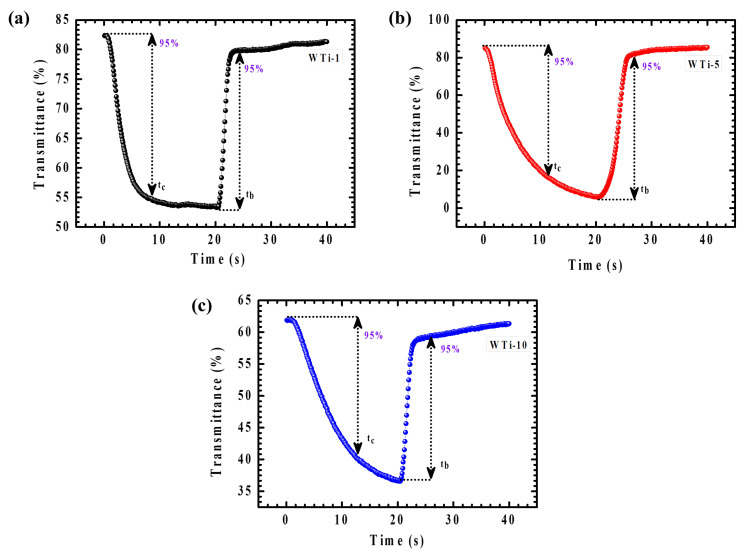
(**a**–**c**) In situ transmittance cycling stability for WTi-1,WTi-5, and WTi-10 heterostructure thin films.

The dynamic EC response of WTi-1, WTi-5, and WTi-10 heterostructure thin films was thoroughly assessed by analyzing their amplified transmittance spectra during the colored and bleached states over a 40 s switching cycle, as shown in Figure 10a–c. Real-time transmittance measurements enabled an accurate determination of the coloration time (t_c_) and bleaching time (t_b_), defined as the time required to achieve 95% of the full transmittance modulation. This kinetic evaluation provides critical insights into the materials’ charge-transfer dynamics, ion diffusion behavior, and their ability to reach steady-state optical modulation under an applied potential. As reported in Table 2, the WTi-5 electrode demonstrated a coloration time of 15.4 s and a bleaching time of 6.2 s. The relatively faster bleaching process is attributed to the superior electronic conductivity of the tungsten bronze and titanium bronze phases present in the bleached state, which facilitates efficient electron transport across the electrode. In contrast, the coloration process is predominantly governed by the intercalation of Li⁺ ions into the electrode structure, a process limited by ionic diffusion and charge-transfer resistance at the electrode–electrolyte interface. The moderate switching times of the WTi-5 film highlight its rapid ion diffusion capability, efficient charge-transfer kinetics, and low internal resistance, confirming its potential for high-performance EC applications in energy-efficient smart window technologies [29].

**Figure 11:**
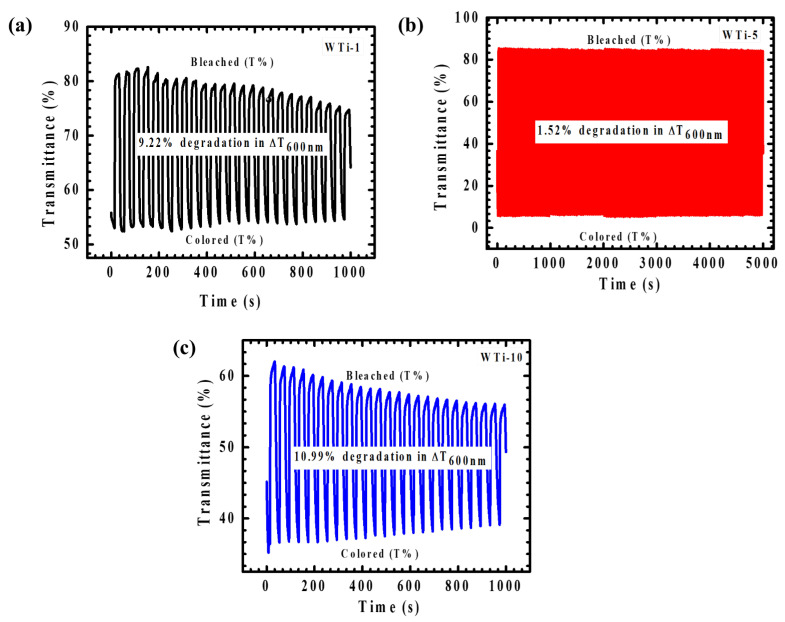
(**a**–**c**) Plot of coloration/bleaching response time for all WTi heterostructure thin films.

The cycling stability of WTi heterostructure electrodes, a critical performance metric for EC applications, was systematically evaluated to assess their long-term durability and functional reliability. As shown in Figure 11, the optimized WTi-5 thin film exhibited outstanding cycling endurance, as evidenced by in situ transmittance monitoring at 600 nm. Over an extended testing period of 5000 s, WTi-5 maintained its optical performance, with only a minimal degradation of 1.52%, highlighting its robust structural integrity and superior EC functionality. The exceptional cycling stability of WTi-5 (Figure 11c) can be attributed to the synergistic combination of WO_3_ and TiO_2_, whose high structural stability provides a resilient framework that facilitates efficient and reversible ion intercalation/deintercalation without significant electrochemical degradation. Moreover, the well-adhered and uniform surface morphology of WTi-5 effectively mitigates the development of mechanical stress and suppresses structural fatigue during continuous cycling, thereby minimizing ion trapping and maintaining a consistent optical modulation. In contrast, WTi-1 (Figure 11a) and WTi-10 (Figure 11b) demonstrated markedly inferior cycling stability, with optical modulation degradations of 9.22% and 10.99%, respectively, after only 1000 s of operation. These films exhibited a significant performance instability, particularly in the case of WTi-10, which showed rapid deterioration beyond the 500 s mark. The pronounced decline in cycling stability in these samples is primarily associated with inefficient ion transport kinetics and a suboptimal surface morphology, which facilitate ion trapping and hinder reversible switching between colored and bleached states [30,31].

To evaluate the practical competitiveness of the WTi-5 heterostructure, a comparative analysis was performed against various WO_3_/TiO_2_ and other materials based on EC systems reported in the literature, as summarized in Table 3. The WTi-5 film demonstrated a high optical modulation of 82.16% at 600 nm, a notable coloration efficiency of 128.93 cm^2^/C, and excellent cycling stability, with minimal degradation over 5000 s of continuous operation. Although certain configurations, such as the TiO_2_/WO_3_/TiO_2_ multilayer fabricated by magnetron sputtering, report higher coloration efficiencies, they typically require high-vacuum deposition techniques that involve elevated fabrication costs, complex instrumentation, and limited compatibility with large-area or flexible substrates. In contrast, the methodology employed in the present work is entirely solution-based, involving low-temperature electrodeposition and hydrothermal growth. These techniques are not only cost-effective and environmentally benign but also inherently scalable using conventional batch or continuous reactor systems. The inclusion of polyvinylpyrrolidone during WO_3_ deposition further improves the film’s uniformity and electrochemical accessibility, without introducing additional complexity in terms of its synthesis. This combination of a strong EC performance, excellent durability, and scalable, low-cost processing highlights the WTi-5 heterostructure as a promising and practically viable alternative to conventional WO_3_-based EC materials for next-generation smart window applications.

## 6. Electrochromic Device Performance

**Figure 12:**
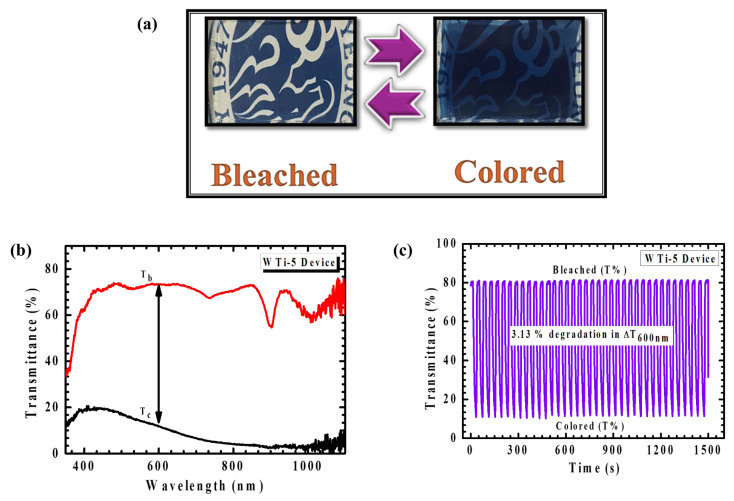
(**a**) Photograph of the WTi-5 heterostructure thin film device in the bleached and colored state. (**b**) In situ transmittance and (**c**) long-term stability of WTi-5 device.

The realization of a functional ECD is essential for translating material-level performance into viable smart-window technologies. In this study, a large-area ECD was assembled using the optimized WO_3_/TiO_2_ (WTi-5) heterostructure film as the electrochromically active working electrode. The WTi-5 film was fabricated by sequential deposition, beginning with the electrodeposition of a WO_3_ layer onto a fluorine-doped tin oxide (FTO) substrate, followed by the hydrothermal growth of a TiO_2_ overlayer for 5 h. The device adopted a conventional sandwich architecture comprising the WTi-5 film as the working electrode, LiClO_4_–PC electrolyte, and a transparent FTO counter electrode. This configuration was selected to facilitate efficient ionic conductivity and maximize electrochemical contact between the electrodes and the electrolyte medium. Photographs of the assembled device (Figure 12a) reveal its rapid and visually distinct transition between a transparent (bleached) state and a deeply colored blue state under an applied potential bias, reflecting the strong EC response of the WTi-5 film. The sharp optical transition underscores the fast redox kinetics and reversible ion intercalation facilitated by the heterostructured interface. In situ transmittance spectra recorded over the 350–1100 nm range (Figure 12b) further validate this behavior, with a significant transmittance modulation of 61.83% at 600 nm, indicating excellent dynamic control over the passage of light in the visible spectrum. This degree of modulation is particularly desirable for smart window applications where both energy savings and visual comfort are critical. The device’s optical performance under real-time operating conditions demonstrated strong consistency with results obtained in standard three-electrode configurations, confirming the practical translatability of the WTi-5 heterostructure to full device integration. The synergy between the amorphous WO_3_ layer, which promotes a high ionic mobility, and the crystalline TiO_2_ overlayer, which stabilizes the interfacial charge dynamics, plays a decisive role in enabling efficient optical switching and sustaining structural integrity during cycling. Cycling stability was assessed through repetitive coloration–bleaching tests over 1500 s, as shown in Figure 12c. The device retained an excellent modulation stability throughout the initial cycles, with only a minor decrease of 3.13% in optical contrast observed toward the end of the test window. This small decline points to a minor interfacial degradation under prolonged operation, though the overall EC performance remained robust. The results emphasize not only the scalability of the heterostructured film but also its reliability under extended functional stress. Taken together, these findings confirm the WO_3_/TiO_2_ heterostructure synthesized in this work as a high-potential candidate for scalable, energy-efficient EC applications. Its carefully engineered layered architecture offers both a fast optical response and endurance, validating the importance of interface design and synthesis optimization in achieving next-generation smart window technologies.

## 7. Conclusions

This study presents the successful development and characterization of a WO_3_/TiO_2_ heterostructure for enhanced EC functionality. By depositing an amorphous WO_3_ layer via electrodeposition and subsequently growing a crystalline TiO_2_ overlayer through hydrothermal synthesis, a bilayer heterostructure was achieved that integrates complementary material properties within a unified architecture. The systematic variation of TiO_2_ growth time revealed its critical role in tuning crystallinity, interface quality, and electrochemical behavior. Among all the configurations, the WTi-5 sample exhibited optimal performance, delivering a high coloration efficiency (128.93 cm^2^/C), fast switching response (15.4 s coloration, 6.2 s bleaching), and strong optical modulation (82.16%). A corresponding large-area electrochromic device based on WTi-5 also demonstrated excellent durability and retained its performance over extended cycling, confirming the practical applicability of this heterostructure.

Furthermore, the synthesis approach presented in this study is inherently scalable and industrially relevant. Both the electrodeposition of WO_3_ and the hydrothermal growth of TiO_2_ are solution-based and cost-effective methods that utilize commercially available precursors. The electrodeposition process is highly controllable and suitable for large-area conductive glass substrates, making it compatible with roll-to-roll or batch processing techniques. Similarly, the hydrothermal treatment operates at relatively low temperatures and can be scaled using high-capacity autoclaves or continuous-flow reactors. Importantly, all the processing steps, including post-annealing, are aligned with standard manufacturing protocols, thereby ensuring the feasibility of scaling this method for the fabrication of EC coatings over architectural glass and flexible devices. The synergy between the amorphous WO_3_ base layer and the crystalline TiO_2_ overlayer facilitates efficient ion transport and stable interfacial dynamics. These results not only advance the understanding of layered electrochromic systems but also demonstrate the potential of WO_3_/TiO_3_ heterostructures for scalable, cost-effective, and energy-efficient smart window technologies.

## Figures and Tables

**Table 1:** Summary of key XRD diffraction peaks and XPS binding energies for the WO_3_/TiO_2_ heterostructure.

Technique	Feature	Position (2θ or BE)	Assignment	Phase/Oxidation State
XRD	(110)	27.4°	TiO_2_ crystal plane	Rutile TiO_2_ (JCPDS 01-088-1175)
XRD	(101)	36.1°	TiO_2_ crystal plane	Rutile TiO_2_
XRD	(211)	54.3°	TiO_2_ crystal plane	Rutile TiO_2_
XRD	(310)	69.0°	TiO_2_ crystal plane	Rutile TiO_2_
XRD	Broad hump	20–27°	Amorphous background (WO_3_ base layer)	Amorphous WO_3_
XPS	W 4f_7/2_	35.7 eV	Spin–orbit peak of tungsten	W^6+^ in WO_3_
XPS	W 4f_5/2_	37.8 eV	Spin–orbit peak of tungsten	W^6+^ in WO_3_
XPS	Ti 2p_3/2_	458.5 eV	Spin–orbit peak of titanium	Ti^4+^ in TiO_2_
XPS	Ti 2p_1/2_	464.2 eV	Spin–orbit peak of titanium	Ti^4+^ in TiO_2_
XPS	O 1 s (lattice oxygen)	530.2 eV	Lattice oxygen in metal oxides	O^2-^
XPS	O 1 s (surface species)	531.5 eV	Surface hydroxyls/chemisorbed O	-

**Table 2:** Evaluation of electrochromic measurements of WTi heterostructure thin films.

Sample Name	Charge Intercalation (Qi) (C/cm^2^)	Charge Deintercalation (Qdi) (C/cm^2^)	Reversibility (%)	Coloration Time (sec) (t_c_)	Bleaching Time (sec) (t_b_)	T_b_%	T_C_%	Optical Modulation (ΔT_600nm_%)	Optical Density (ΔOD)	Coloration Efficiency (cm^2^/C)
WTi-1	0.051	0.050	98.03	8.7	3.1	81.37	53.02	31.07	1.53	30.00
**WTi-5**	**0.066**	**0.065**	**98.48**	**15.4**	**6.2**	**86.32**	**4.16**	**82.16**	**6.06**	**128.93**
WTi-10	0.047	0.046	97.80	14.1	5.8	66.05	35.47	30.58	1.20	25.53

**Table 3:** Comparative assessment of electrochromic measurements of WTi-5 heterostructure thin film with the existing literature.

Material	Method	Morphology	ΔT%	Coloration Efficiency (cm^2^/C)	Coloration Time (t_c_; sec)	Bleaching Time (t_b_; sec)	Stability	Ref.
TiO_2_@WO_3_	Hydrothermal and electrodeposition	Nanorods	57.2	67.5	5.4	6.4	10,000 cycles	[12]
TiO_2_/WO_3_/TiO_2_	Magnetron sputtering	Compact and dense nanogranules	94.72	479.3	5.6	2.9	7000 cycles	[13]
TiO_2_-WO_3_	Doctor blade and electrodeposition	Nanopores	-	55.1	-	-	-	[15]
TiO_2_/WO_3_	Spin coating	Nanograins	75	-	-	-	-	[16]
TiO_2_/WO_3_	Hydrothermal	Nanotrees	79.5	443.4	1.9	14.8	10,000	[17]
TiO_2_–Co_3_O_4_	Hydrothermal	Nanorods	-	91	-	-	-	[18]
Nb_2_O_5_	Solvothermal	Spherical particles	69	85.9	10.6	0.7	400 cycles	[30]
NiO	E-beam evaporation	granular	66	55	3.6	1.4	200 cycles	[32]
V_2_O_5_	Crystal-assisted electrodeposition	Nanorod	27.43	25.2	8.8	9.3	-	[33]
NiO	Sol–gel dip coating	Nanorod	68	-	5.6	2.3	1200 cycles	[34]
**WO_3_/TiO_2_**	**Electrodeposition and hydrothermal**	**Spherical**	**82.16**	**128.93**	**15.4**	**6.2**	**5000**	** This Work **

## Data Availability

The raw data supporting the conclusions of this article will be made available by the authors on request.

## References

[1] Li B., Dang J., Zhuang Q., Lv Z. (2022). Recent Advances in Inorganic Electrochromic Materials from Synthesis to Applications: Critical Review on Functional Chemistry and Structure Engineering. Chem.-Asian J..

[2] Reyes-Gil K.R., Stephens Z.D., Stavila V., Robinson D.B. (2015). Composite WO_3_/TiO_2_ Nanostructures for High Electrochromic Activity. ACS Appl. Mater. Interfaces.

[3] Afik N., Murugesan S., Shreteh K., Fridman H., Hijaze Y., Volokh M., Mokari T. (2024). Synthesis of Ultrathin Alloy (Mo, V)-Tungsten-Oxide Nanowires: Implications for Electrochromic and Supercapacitor Applications. ACS Appl. Nano Mater..

[4] Amate R.U., Morankar P.J., Teli A.M., Bhosale M.K., Ahir N.A., Jeon C.W. (2025). Interface-Centric Strategies in Nb_2_O_5_/MoS_2_ Heterostructure: Leveraging Synergistic Potential for Dual-Function Electrochromic Energy Storage. Chem. Eng. J..

[5] Gu C., Jia A.B., Zhang Y.M., Zhang S.X.A. (2022). Emerging Electrochromic Materials and Devices for Future Displays. Chem. Rev..

[6] Lee J.H., Kim H., Hwang J.Y., Chung J., Jang T.M., Seo D.G., Gao Y., Lee J., Park H., Lee S. (2020). 3D Printed, Customizable, and Multifunctional Smart Electronic Eyeglasses for Wearable Healthcare Systems and Human-Machine Interfaces. ACS Appl. Mater. Interfaces.

[7] Zhang W., Li H., Hopmann E., Elezzabi A.Y. (2020). Nanostructured Inorganic Electrochromic Materials for Light Applications. Nanophotonics.

[8] Gao M., Bao Y., Qian Y., Deng Y., Li Y., Chen G. (2018). Porous Anatase-TiO_2_(B) Dual-Phase Nanorods Prepared from in Situ Pyrolysis of a Single Molecule Precursor Offer High Performance Lithium-Ion Storage. Inorg. Chem..

[9] Fan X., Pan M., Li X., Kong L., Kuchmizha A., Xu H. (2024). Research Progress of MOF Electrochromic Materials. Resour. Chem. Mater..

[10] Wen R.T., Granqvist C.G., Niklasson G.A. (2015). Eliminating Degradation and Uncovering Ion-Trapping Dynamics in Electrochromic WO_3_ Thin Films. Nat. Mater..

[11] Morankar P.J., Amate R.U., Teli A.M., Beknalkar S.A., Jeon C.W. (2024). Exploring Electrochromic Performance via Layered Deposition of Tungsten Oxide on Niobium Oxide Composite Electrode. J. Power Sources.

[12] Cai G.F., Zhou D., Xiong Q.Q., Zhang J.H., Wang X.L., Gu C.D., Tu J.P. (2013). Efficient Electrochromic Materials Based on TiO_2_@WO_3_ Core/Shell Nanorod Arrays. Sol. Energy Mater. Sol. Cells.

[13] Lv Z., Yang D., Mo J., Jin Z., Chang S. (2024). Construction of TiO_2_/WO_3_/TiO_2_ Double Heterojunction Films for Excellent Electrochromic Performance. Sci. Rep..

[14] Nah Y.C., Ghicov A., Kim D., Berger S., Schmuki P. (2008). TiO_2_-WO_3_ Composite Nanotubes by Alloy Anodization: Growth and Enhanced Electrochromic Properties. J. Am. Chem. Soc..

[15] Ninh D.H., Thao T.T., Dinh N.N., Long P.D. (2014). Characterization of Structural and Electrochromic Properties of Nanocomposite (TiO_2_/WO_3_) Films. Commun. Phys..

[16] Hsu C.S., Lin C.K., Chan C.C., Chang C.C., Tsay C.Y. (2006). Preparation and Characterization of Nanocrystalline Porous TiO_2_/WO_3_ Composite Thin Films. Thin Solid Film..

[17] Zhao L., Cai Z., Wang X., Liao W., Huang S., Ye L., Fang J., Wu C., Qiu H., Miao L. (2023). Constructed TiO_2_/WO_3_ Heterojunction with Strengthened Nano-Trees Structure for Highly Stable Electrochromic Energy Storage Device. J. Adv. Ceram..

[18] Mishra S., Yogi P., Sagdeo P.R., Kumar R. (2018). TiO_2_-Co_3_O_4_ Core-Shell Nanorods: Bifunctional Role in Better Energy Storage and Electrochromism. ACS Appl. Energy Mater..

[19] Morankar P.J., Amate R.U., Chavan G.T., Teli A.M., Dalavi D.S., Jeon C.W. (2023). Improved Electrochromic Performance of Potentiostatically Electrodeposited Nanogranular WO_3_ Thin Films. J. Alloys Compd..

[20] Amate R.U., Morankar P.J., Teli A.M., Beknalkar S.A., Jeon C.W. (2025). Synergistic Design of Processable Nb_2_O_5_-TiO_2_ Bilayer Nanoarchitectonics: Enabling High Coloration Efficiency and Superior Stability in Dual-Band Electrochromic Energy Storage. J. Colloid Interface Sci..

[21] Patrocinio A.O.T., Paula L.F., Paniago R.M., Freitag J., Bahnemann D.W. (2014). Layer-by-Layer TiO_2_/WO_3_ Thin Films As E Ffi Cient Photocatalytic Self- Cleaning Surfaces. ACS Appl. Mater. Interfaces.

[22] Erdem B., Hunsicker R.A., Simmons G.W., David Sudol E., Dimonie V.L., El-Aasser M.S. (2001). XPS and FTIR Surface Characterization of TiO_2_ Particles Used in Polymer Encapsulation. Langmuir.

[23] van Ommen J.R., Valverde J.M., Pfeffer R. (2012). Fluidization of Nanopowders: A Review. J. Nanoparticle Res..

[24] Kim J., Ong G.K., Wang Y., Leblanc G., Williams T.E., Mattox T.M., Helms B.A., Milliron D.J. (2015). Nanocomposite Architecture for Rapid, Spectrally-Selective Electrochromic Modulation of Solar Transmittance. Nano Lett..

[25] Wu W., Wang M., Ma J., Cao Y., Deng Y. (2018). Electrochromic Metal Oxides: Recent Progress and Prospect. Adv. Electron. Mater..

[26] Nguyen T.H.Q., Eberheim F., Göbel S., Cop P., Eckert M., Schneider T.P., Gümbel L., Smarsly B.M., Schlettwein D. (2022). Enhancing the Spectroelectrochemical Performance of WO_3_ Films by Use of Structure-Directing Agents during Film Growth. Appl. Sci..

[27] Koilraj P., Takemoto M., Tokudome Y., Bousquet A., Prevot V., Mousty C. (2020). Electrochromic Thin Films Based on NiAl Layered Double Hydroxide Nanoclusters for Smart Windows and Low-Power Displays. ACS Appl. Nano Mater..

[28] Amate R.U., Morankar P.J., Jeon C.W. Tuning Electrochromic Behavior through Surfactant-Mediated Structural Modifications in Nb_2_O_5_/WO_3_ Heterostructures. Ceram. Int..

[29] Zhang S., Cao S., Zhang T., Fisher A., Lee J.Y. (2018). Al^3+^ Intercalation/de-Intercalation-Enabled Dual-Band Electrochromic Smart Windows with a High Optical Modulation, Quick Response and Long Cycle Life. Energy Environ. Sci..

[30] Yu C., Ma D., Wang Z., Zhu L., Guo H., Zhu X., Wang J. (2021). Solvothermal Growth of Nb_2_O_5_ Films on FTO Coated Glasses and Their Electrochromic Properties. Ceram. Int..

[31] Lu H.C., Zydlewski B.Z., Tandon B., Shubert-Zuleta S.A., Milliron D.J. (2022). Understanding the Role of Charge Storage Mechanisms in the Electrochromic Switching Kinetics of Metal Oxide Nanocrystals. Chem. Mater..

[32] Pereira S., Gonçalves A., Correia N., Pinto J., Pereira L., Martins R., Fortunato E. (2014). Electrochromic Behavior of NiO Thin Films Deposited by E-Beam Evaporation at Room Temperature. Sol. Energy Mater. Sol. Cells.

[33] Tong Z., Zhang X., Lv H., Li N., Qu H., Zhao J., Li Y., Liu X.Y. (2015). From Amorphous Macroporous Film to 3D Crystalline Nanorod Architecture: A New Approach to Obtain High-Performance V_2_O_5_ Electrochromism. Adv. Mater. Interfaces.

[34] Purushothaman K.K., Muralidharan G. (2009). The Effect of Annealing Temperature on the Electrochromic Properties of Nanostructured NiO Films. Sol. Energy Mater. Sol. Cells.

